# Intravenous Thrombolysis in Acute Ischemic Stroke: A Prognostic Prediction Model and the Role of Ischemic Core Growth Rate

**DOI:** 10.1111/cns.70589

**Published:** 2025-09-04

**Authors:** Yipeng Yu, Lulu Zhang, Qi Fang

**Affiliations:** ^1^ Department of Neurology The First Affiliated Hospital of Soochow University Suzhou China; ^2^ Department of Neurology Dushu Lake Hospital Affiliated to Soochow University Suzhou China

**Keywords:** cerebrovascular circulation, ischemic stroke, nomograms, prognosis, thrombolytic therapy

## Abstract

**Aims:**

The tissue window is increasingly recognized in guiding reperfusion therapy beyond the standard time window in acute ischemic stroke (AIS). This study aims to develop a nomogram incorporating an ischemic core growth rate index to provide individualized prediction of neurological outcomes in AIS patients who received intravenous thrombolysis (IVT).

**Methods:**

A retrospective study was conducted at the First Affiliated Hospital of Soochow University (2016–2023). A lasso‐logistic method was employed for variable selection and model construction. The performance of the model was evaluated using the receiver operating characteristic curve, calibration curve, decision curve analysis, and compared with a conventional indexed one.

**Results:**

The study cohort comprised 553 patients with favorable outcomes (median ischemic core growth rate: 1.4 [0.5, 4.1] mL/h) and 198 patients with poor outcomes (median ischemic core growth rate: 5.7 [1.1, 14.2] mL/h). The nomogram included diabetes, TOAST classification, ischemic core growth rate, neutrophil count, direct bilirubin, and NIHSS score at admission. It achieved an AUC of 0.882 (95% CI: 0.855–0.908), outperforming the conventional indexed one. Calibration showed good agreement between predicted and observed outcomes (Hosmer–Lemeshow *p* = 0.851).

**Conclusion:**

Ischemic core growth rate strongly correlates with neurological prognosis in AIS. This nomogram offers reliable predictions for IVT outcomes.

## Graphical abstract

In this study, we introduced an *ischemic growth rate* index and examined its value in outcome prediction. Additionally, we developed a nomogram to provide individual prediction of neurological outcome.

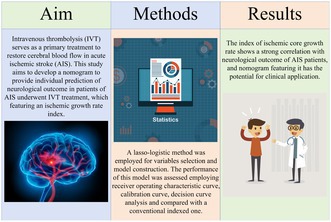

## 1 Introduction

Acute ischemic stroke (AIS) is a major cerebrovascular event with a high risk of mortality and disability [1]. Despite the significant efficacy that intravenous thrombolysis (IVT) has achieved in rescuing ischemic tissue and reversing neurological deficits [2, 3], a subset of patients exhibits limited neurological improvement or even clinical deterioration [4, 5, 6, 7]. For this reason, there is a necessity in predicting the functional outcome of AIS patients postoperatively to determine a further need for therapeutic adjustment or refinement [8, 9].

With the highlight of the temporal factor in the treatment of AIS, a concept of ischemic growth rate has been proposed in previous studies [10, 11]. As a composite indicator that integrates the temporal factor with imaging information, this novel index has been found to be in correlation with collateral circulation status and holds value in estimating the final infarct volume [12, 13]. However, the prognostic prediction value of this ischemic growth rate index still needs further exploration.

This study employed a large cohort with high‐dimensional variables of AIS patients who underwent IVT treatment at the First Affiliated Hospital of Soochow University from 2016 to 2023. Our aim is to investigate the predictive value of the ischemic growth rate index for neurological outcomes in AIS. Additionally, we aim to develop a nomogram to provide an individual risk of poor neurological outcome in a convenient manner.

## 2 Materials and Methods

### 2.1 Study Design and Patient Selection

This retrospective study was conducted in the Department of Neurology, the First Affiliated Hospital of Soochow University. Approval was granted by the Ethics Committee of the First Affiliated Hospital of Soochow University (No. 2024656) and the requirement of written informed consent was waived by the Ethics Committee. All data involving patients' personal information was removed from the entire dataset before analysis.

Our study included the medical record of 1044 AIS patients who underwent IVT therapy within 24 h after stroke onset. The inclusion criteria for patients are as follows: (1) age of at least 18 years; (2) baseline modified Rankin Scale (mRS) score of 0 to 1 [14]; (3) AIS confirmed typically by neuroimaging of computed tomography perfusion (CTP) and non‐contrast computed tomography (NCCT) in patients with neurological impairment [15, 16]; (4) receipt of intravenous rt‐PA thrombolytic therapy within 24 h of the last known well state; (5) evidence of salvageable brain tissue as an initial ischemic core volume < 70 mL and a ratio of ischemic tissue volume to initial infarct volume of at least 1.8 [17].

To ensure the accuracy of our study, we also adopted several exclusion criteria during the enrollment to optimize the representativeness of the samples. The exclusion criteria are as follows: (1) intracranial hemorrhage (ICH) confirmed by imaging methods; (2) rapid improvement of symptoms, as judged by the neurologists; (3) pre‐stroke mRS score higher than 2; (4) contraindications for contrast agent imaging; (5) patients receiving any endovascular interventions other than IVT [18]; (6) any terminal illness with an expected survival of no more than 1 year; (7) patients with severe renal dysfunction (serum creatinine > 133 μmol/L) [19, 20]; (8) patients with coagulopathy (INR > 1.7) [21]; (9) patients with severe systemic inflammation (white blood cell > 12 × 10^9^/L or < 4 × 10^9^/L) [22, 23]; (10) recent history or clinical manifestations of subarachnoid hemorrhage, arteriovenous malformations, aneurysms, or brain tumor; (11) clinically significant hypoglycemia; (12) uncontrolled hypertension, defined as a systolic blood pressure (SBP) > 185 mmHg or diastolic blood pressure (DBP) > 110 mmHg on at least two independent measurements taken at least 10 min apart, or the need for aggressive treatment to reduce blood pressure within these limits [24].

**FIGURE 1:**
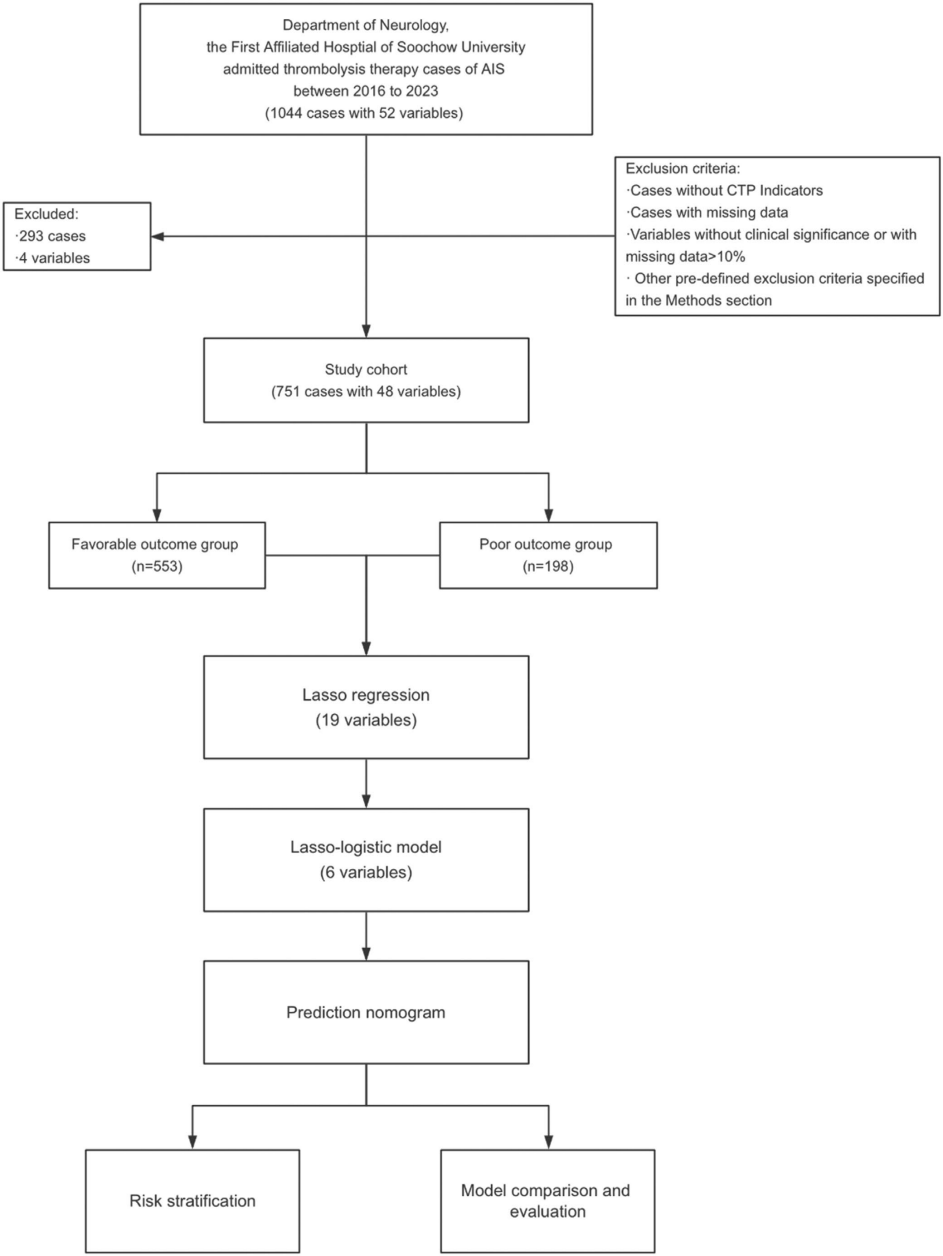
Study flowchart. CTP, computed tomography perfusion.

This study also excluded indicators that could directly lead to a poor outcome, such as hypoperfusion volume [25], as well as those without potential clinical significance, like whether craniectomy and decompression were performed [26]. The cases filtration and study flow chart are illustrated in Figure 1. This study strictly adhered to the principles of the Declaration of Helsinki [27], ensuring the protection of research ethics and patient rights.

### 2.2 Demographic and Laboratory Parameters

The demographical and laboratory parameters are obtained from the electronic medical record system, which include: (1) basic information and pre‐admission medical history: age, gender, blood pressure, hyperlipidemia, smoking, drinking, diabetes mellitus (DM), atrial fibrillation (AF), history of ICH, previous stroke, myocardial infarction (MI), and status of antiplatelet therapy (APT), etc. (2) stroke‐related information: the TOAST classification, hypoperfusion volume, ischemic core volume, penumbra volume, the onset‐to‐treatment (OTT) time, post‐thrombolysis hemorrhagic transformation (HT), etc. (3) laboratory parameters: white blood cells (WBC), monocytes (MO), lymphocytes (LY), neutrophils (NE), platelets (PLT), red blood cell distribution width (RDW), mean platelet volume (MPV), platelet distribution width (PDW), prothrombin time (PT), activated partial thromboplastin time (APTT), international normalized ratio (INR), aspartate aminotransferase (AST), alanine aminotransferase (ALT), total bilirubin (TBIL), direct bilirubin (DBIL), indirect bilirubin (IBIL), uric acid (UA), serum creatinine (Cr), total cholesterol (TC), triglycerides (TG), high‐density lipoprotein (HDL), low‐density lipoprotein (LDL), fibrinogen (Fib), and homocysteine (HCY); neurological deficits were evaluated using the National Institutes of Health Stroke Scale (NIHSS) score upon admission.

### 2.3 Reperfusion Therapy and Radiological Assessment

Upon hospital admission, patients first underwent multimodal CT examinations in the imaging core laboratory, including NCCT and CTP imaging. These neuroimaging findings were independently reviewed by at least two neuroradiologists to establish diagnosis and guide treatment planning [28]. Then, a selected cohort received a standard dose of rt‐PA (Actilyse, Boehringer Ingelheim Pharma, Germany) treatment subsequently [29].

The CTP imaging data were acquired from the MIStar software (Apollo Medical Imaging Technology, Australia). This software automatically calculates the volumes of hypoperfusion, ischemic core, and penumbra based on the preset parameters and thresholds. We adopted the default parameters: ischemic core defined as the region with a cerebral blood flow < 30% within the hypoperfusion lesion, while penumbra represents a mismatch between the hypoperfusion lesion and ischemic core [30, 31, 32]. The calculation of the growth rate index assumes that ischemic core increases linearly following a cerebrovascular event [33]. Specifically, the computation of this index derived from this formula:

Ischemic Core Growth Rate=Ischemic Core Volume onCTP/OTTtime

We further classified it into three levels: low, medium, and high, based on terciles [33]. Additionally, the penumbra growth rate index is calculated using the same methodology.

### 2.4 Outcome Evaluation and Patient Groups

The functional outcome was defined as the neurological status of patients at 90 days after stroke assessed using the mRS score [34]. Previous studies have commonly defined an mRS score of 0 to 2 as an indicator of functional independence, while a score of 3 to 6 typically indicates poor prognosis [35, 36].

Therefore, we subsequently divided the study cohort into two groups based on their mRS scores at 90 days for further analysis, with a score of 0 to 2 designated as the favorable outcome group and a score of 3 to 6 as the poor outcome group. Table 1 lists the detailed information between these two groups.

**TABLE 1:** Comparison of demographic and clinical characteristics between patients with favorable and poor outcomes.

Variables	Favorable outcome group (*n* = 553)	Poor outcome group (*n* = 198)	*p*
Sex, *n* (%)			0.516^b^
Female	176 (31.8%)	71 (35.9%)	
Male	377 (68.2%)	127 (64.1%)	
Age median (IQR), years	65.0 (56.0, 74.0)	72.0 (62.3, 78.0)	< 0.001^a,^***
Systolic blood pressure median (IQR), mmHg	155.0 (139.0, 174.0)	156.0 (138.0, 171.0)	0.777^a^
Diastolic blood pressure median (IQR), mmHg	88.0 (79.0, 96.0)	87.0 (78.3, 98.0)	0.991^a^
Comorbidities, *n* (%)			
History of intracranial hemorrhage	46 (8.3%)	46 (23.2%)	< 0.001^b,^***
Hypertension	386 (69.8%)	143 (72.2%)	0.583^b^
Diabetes mellitus	115 (20.8%)	60 (30.3%)	0.009^b,^**
Smoking	135 (24.4%)	42 (21.2%)	0.126^b^
Drinking	103 (18.6%)	27 (13.6%)	0.138^b^
Atrial fibrillation	84 (15.2%)	68 (34.3%)	< 0.001^b,^***
Antiplatelet therapy	46 (8.3%)	21 (10.6%)	0.410^b^
Hyperlipidemia	42 (7.6%)	16 (8.1%)	0.948^b^
Myocardial infarction	9 (1.6%)	8 (4.0%)	0.089^c^
History of stroke	56 (10.1%)	19 (9.6%)	0.940^b^
Neuroimaging information			
Hypoperfusion volume median (IQR), mL	39.0 (14.0, 81.0)	74.9 (15.0, 148.8)	< 0.001^a,^***
Ischemic core volume median (IQR), mL	4.0 (1.9, 11.0)	15.0 (4.0, 45.3)	< 0.001^a,^***
Penumbra volume median (IQR), mL	34.0 (13.0, 67.0)	68.5 (14.3, 113.0)	< 0.001^a,^***
Onset‐to‐treatment time median (IQR), h	3.0 (2.1, 4.0)	3.2 (2.3, 4.0)	0.118^a^
Ischemic core growth rate median (IQR), mL/h	1.4 (0.5, 4.1)	5.7 (1.1, 14.2)	< 0.001^a,^***
Low, *n* (%)	417 (75.4%)	79 (39.9%)	< 0.001^b,^***
Medium, *n* (%)	89 (16.1%)	44 (22.2%)	< 0.001^b,^***
High, *n* (%)	47 (8.5%)	75 (37.9%)	< 0.001^b,^***
Penumbra growth rate median (IQR), mL/h	10.8 (4.2, 25.9)	23.1 (10.1, 40.1)	< 0.001^a,^***
Low, *n* (%)	373 (67.5%)	62 (31.3%)	< 0.001^b,^***
Medium, *n* (%)	102 (18.4%)	58 (29.3%)	< 0.001^b,^***
High, *n* (%)	78 (14.1%)	78 (39.4%)	< 0.001^b,^***
TOAST classification, *n* (%)			
LAA	255 (46.1%)	111 (56.1%)	0.020^b,^*
CE	80 (14.5%)	61 (30.8%)	< 0.001^b,^***
SAA	154 (27.8%)	15 (7.6%)	< 0.001^b,^***
SOE	16 (2.9%)	1 (0.5%)	0.054^c^
SUE	48 (8.7%)	10 (5.0%)	0.137^b^
Hemorrhagic transformation, *n* (%)	90 (16.3%)	26 (13.1%)	0.471^b^
Laboratory parameters			
Total bilirubin median (IQR), μmol/L	16.1 (12.4, 21.1)	19.5 (15.0, 26.3)	< 0.001^a,^***
Direct bilirubin median (IQR), μmol/L	5.2 (3.9, 6.9)	6.5 (5.0, 9.1)	< 0.001^a,^***
Indirect bilirubin median (IQR), μmol/L	10.8 (8.4, 13.9)	13.1 (9.2, 17.2)	< 0.001^a,^***
Alanine aminotransferase median (IQR), U/L	21.0 (15.2, 29.0)	20.0 (16.2, 31.0)	0.482^a^
Aspartate aminotransferase median (IQR), U/L	23.0 (18.4, 29.0)	27.0 (21.0, 32.0)	< 0.001^a,^***
Glucose median (IQR), mmol/L	6.7 (5.7, 8.4)	7.4 (6.2, 9.8)	< 0.001^a,^***
White blood cells median (IQR), ×10^9^/L	7.5 (6.3, 9.2)	8.3 (6.7, 10.7)	< 0.001^a,^***
Monocytes median (IQR), ×10^9^/L	0.4 (0.3, 0.5)	0.4 (0.3, 0.6)	0.080^a^
Lymphocytes median (IQR), ×10^9^/L	1.8 (1.3, 2.3)	1.4 (1.1, 1.9)	< 0.001^a,^***
Neutrophils median (IQR), ×10^9^/L	4.8 (3.8, 6.7)	6.1 (4.6, 8.2)	< 0.001^a,^***
Red blood cell distribution width median (IQR), %	12.9 (12.5, 13.4)	13.1 (12.7, 13.6)	< 0.001^a,^***
Platelets median (IQR), ×10^9^/L	199.0 (162.0–236.0)	189.5 (157.0–239.8)	0.308^a^
Mean platelet volume median (IQR), fL	9.7 (8.9, 10.5)	9.8 (8.9, 10.6)	0.390^a^
Platelet distribution width median (IQR), %	16.2 (16.0, 16.5)	16.3 (15.9, 16.5)	0.980^a^
High‐density lipoprotein median (IQR), mmol/L	1.0 (0.9, 1.2)	1.1 (0.9, 1.3)	0.007^a,^**
Low‐density lipoprotein median (IQR), mmol/L	2.7 (2.2, 3.4)	2.6 (2.0, 3.2)	0.125^a^
Total cholesterol median (IQR), mmol/L	4.4 (3.8, 5.1)	4.5 (3.7, 5.1)	0.936^a^
Triglycerides median (IQR), mmol/L	1.3 (1.0, 1.8)	1.2 (0.9, 1.6)	0.026^a,^*
Serum creatinine median (IQR), μmol/L	68.0 (58.0, 78.0)	67.0 (57.0, 81.8)	0.879^a^
Uric acid median (IQR), μmol/L	301.0 (242.3, 360.8)	309.1 (246.3, 378.5)	0.336^a^
International normalized ratio median (IQR)	1.0 (0.9, 1.1)	1.0 (0.9, 1.1)	0.040^a,^*
Activated partial thromboplastin time median (IQR), s	31.6 (28.5, 34.9)	32.2 (28.5, 34.7)	0.885^a^
Fibrinogen median (IQR), g/L	3.1 (2.6, 3.6)	3.2 (2.6, 3.7)	0.238^a^
Prothrombin time median (IQR), s	12.9 (12.3, 13.4)	13.1 (12.4, 13.8)	0.016^a,^*
Homocysteine median (IQR), μmol/L	10.6 (8.8, 13.5)	11.4 (9.1, 14.7)	0.022^a,^*
Neurological score			
NIHSS score median (IQR)	4.0 (2.0, 8.0)	13.0 (9.0, 18.0)	< 0.001^a,^***
Modified Rankin Scale score median (IQR)	0.0 (0.0, 1.0)	4.0 (3.0, 5.0)	< 0.001^a,^***

Abbreviations: CE, cardioembolism; IQR, interquartile range; LAA, large artery atherosclerosis; NIHSS, National Institutes of Health Stroke Scale; SAA, small artery occlusion; SOE, other determined etiology; SUE, undetermined etiology; TOAST classification, Trial of Org 10172 in Acute Stroke Treatment classification.

^a^ *p* value derived from Wilcoxon test. ^b^ *p* value derived from chi‐square test. ^c^ *p* value derived from Fisher's exact test. *Indicates a *p* value < 0.05. **Indicates a *p* value < 0.01. ***Indicates a *p* value < 0.001.

### 2.5 Statistical Analysis

All statistical analyses were conducted using R software (version 4.2). Our study cohort included 751 patients with AIS who underwent IVT treatment. Next, we divided the patients into a favorable outcome group (553 patients) and a poor outcome group (198 patients) based on their mRS scores at 90 days. Continuous variables were evaluated for normality using the Shapiro–Wilk test [37], which showed non‐normal distributions for all variables. Therefore, continuous variables were described using median (interquartile range [IQR]), and the statistical difference between groups was analyzed using the Wilcoxon test, whereas categorical variables were presented as count (percentage) and analyzed using the chi‐square test or Fisher's exact test. We employed the Least Absolute Shrinkage and Selection Operator (lasso) regression to screen for predictors from the high‐dimensional dataset, which removes unimportant variables from the model by shrinking their coefficients [38]. The selected variables were then incorporated into a logistic regression analysis to establish the model. Conclusively, a nomogram for predicting the risk of a poor neurological outcome was established, utilizing the lasso‐logistic model as its foundation. This model's design, evaluation, and reporting followed the TRIPOD guidelines for prognostic studies [39].

Furthermore, we determined the risk stratification threshold of the model using Youden's *J* Statistic [40]. To examine the predictive value of this ischemic growth rate model, we also fitted a conventional index model for comparison, which was based on the original CTP indicators and relevant clinical information. Despite using the same study set for modeling, we conducted separate analyses to ensure that each model identified its own independent predictors associated with clinical outcomes, thereby avoiding potential bias that might arise from enforcing identical results between models. Then the likelihood ratio test was employed to evaluate the similarity among models. Meanwhile, indicators such as Akaike Information Criterion (AIC) and *R*‐squared (*R* ^2^) are adopted to evaluate the model fitting. Validation of consistency is conducted using the Hosmer–Lemeshow test and the calibration curve was plotted to visualize the result. The discriminating performance of models was assessed utilizing the area under the curve (AUC) value as well as misclassification error. Additionally, the net benefit of models was evaluated through the application of decision curve analysis (DCA). The clinical impact curve was plotted as well to demonstrate the relationship between prediction and actual situation. Statistical significance was defined as *p* < 0.05.

## 3 Results

### 3.1 Characteristics of Patients

The study cohort comprised 751 AIS patients who underwent IVT therapy. The median ischemic core growth rates of low, medium, and high were 0.4 (0.3, 0.7), 2.5 (1.6, 3.4), and 11.7 (7.5, 20.1) mL/h, respectively.

Subsequently, we divided these patients into two groups based on their mRS scores at 90 days. The favorable outcome group consisted of 377 men and 176 women, with a median age of 65 years. The median growth rate of ischemic core was 1.4 mL/h. The poor outcome group consisted of 127 men and 71 women, with a median age of 72 years. The median growth rate of ischemic core was 5.7 mL/h. Table 1 presents the landscape of patients between these two groups.

### 3.2 Lasso‐Logistic Method for Variable Screening and Model Fitting

**FIGURE 2:**
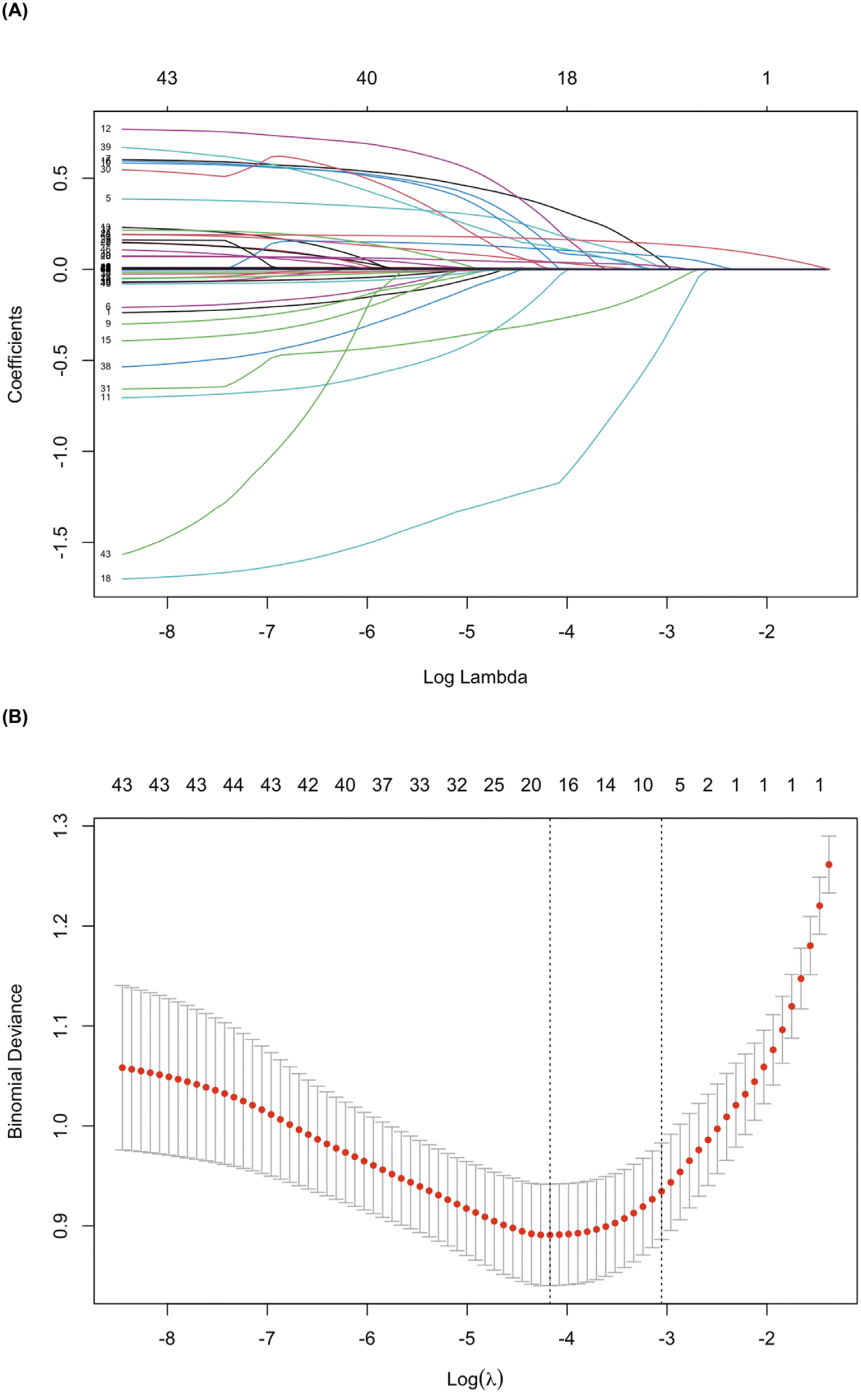
Lasso regression for predictors selection. (A) the curves of coefficients versus log lambda, displaying the shrinking process of factor selection; (B) the validation of the optimal parameter in lasso regression with a plot of binomial deviance versus log(*λ*).

To alleviate the problem of collinearity and avoid overfitting of the predictive model caused by high‐dimensional dataset [41], our study utilized lasso regression for screening the original variables within the study cohort (Figure 2A). The number of variables reduced to 19 when Log (*λ*) reached the minimum mean squared error derived from the internal cross‐validation of the regression (10 *k*‐fold) (Figure 2B).

**FIGURE 3:**
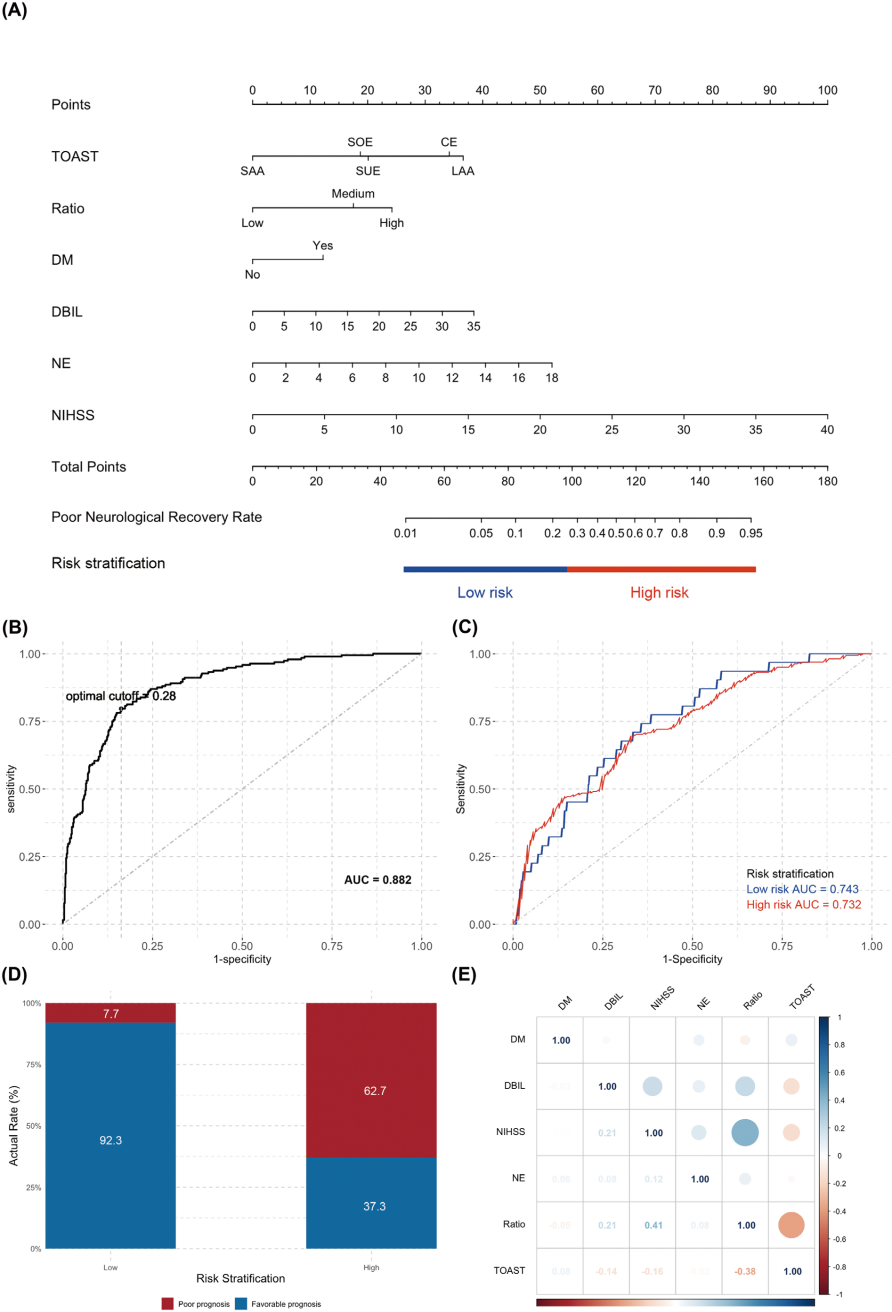
The nomogram for predicting neurological outcome based on lasso‐logistic model. (A) nomogram representing the point assignment for each variable to calculate the predicted probability of poor neurological outcome; the corresponding risk stratifications are labeled at the bottom. (B) the ROC curve and the threshold for risk stratification. (C) ROC curves within each risk group. (D) actual incidence of poor neurological outcome in each risk stratification group. (E) the correlation matrix among variables in the predictive model. AUC, area under the curve; CE, cardioembolism; DBIL, direct bilirubin; DM, diabetes mellitus; LAA, large artery atherosclerosis; NE, neutrophils; NIHSS, National Institutes of Health Stroke Scale score; Ratio, the ischemic core growth rate index; ROC curve, receiver operating characteristic curve; SAA, small artery occlusion; SOE, other determined etiology; SUE, undetermined etiology; TOAST classification, Trial of Org 10172 in Acute Stroke Treatment classification.

Next, we integrated the variables chosen by lasso regression into a binary logistic model for further construction. During the process of modeling, some variables were omitted because of a weak correlation with the response variable. Finally, we included six variables in the lasso‐logistic model; the result showed that all of them were independent predictive factors for the neurological prognosis (*p* < 0.05) (Table 2). We calculated the correlation coefficient matrix among these variables and created a heatmap to visualize the result, which shows no collinearity issue within these variables and indicates successful modeling (Figure 3E).

**TABLE 2:** Independent predictive factors included in the nomogram.

Nomogram	OR (95% CI)	*p*
Ischemic core growth rate (mL/h)		
Low	Ref.	
Medium	1.33 (0.78–2.25)	0.765
High	2.54 (1.45–4.45)	< 0.001***
TOAST classification		
SUE	Ref.	
LAA	1.23 (0.54–2.81)	0.614
CE	0.94 (0.38–2.35)	0.677
SAA	0.26 (0.08–0.83)	0.020*
SOE	0.48 (0.08–2.85)	0.138
Diabetes mellitus	2.31 (1.40–3.81)	< 0.001***
Direct bilirubin (μmol/L)	1.08 (1.01–1.16)	0.017*
Neutrophils (×10^9^/L)	1.17 (1.09–1.27)	< 0.001***
NIHSS score	1.20 (1.15–1.24)	< 0.001***

Abbreviations: CE, cardioembolism; CI, confidence interval; LAA, large artery atherosclerosis; NIHSS, National Institutes of Health Stroke Scale; OR, odds ratio; Ref, reference level; SAA, small artery occlusion; SOE, other determined etiology; SUE, undetermined etiology; TOAST classification, Trial of Org 10172 in Acute Stroke Treatment classification.

*Indicates a *p* value < 0.05. ***Indicates a *p* value < 0.001.

### 3.3 Development of Nomogram and Risk Stratification

Based on the lasso‐logistic model, we developed a nomogram for predicting the neurological outcome of AIS patients (Figure 3A). The nomogram includes six predictive indicators: one medical history indicator (comorbidity of diabetes), two stroke‐related indicators (the TOAST classification and ischemic core growth rate), two laboratory parameters (neutrophil counts and DBIL level), and the patient's NIHSS score at admission.

Considering the feasibility for clinical application, we further stratified the predicted probabilities. By applying the Youden's *J* statistic, we determined the threshold for binary risk stratification in this predictive model, yielding a distinct cutoff value of 0.28, which we designated as the optimal cutoff (Figure 3B). Subsequently, we applied this cutoff value to establish a binary risk stratification category: Low‐ and high‐risk groups, which correspond to a predicted probability of 0.28 or less and a predicted probability > 0.28, respectively. To assess the capacity of this binary risk stratification method in discrimination, we plotted the receiver operating characteristic (ROC) curve within each risk group and calculated the AUC value. The results demonstrated that this binary risk stratification method possessed a good capacity for discrimination (Figure 3C), both among the Low‐risk and the high‐risk groups. Furthermore, we made an inventory of the virtual occurrence of poor neurological outcome across different risk groups and the results showed that this binary method demonstrated good performance of stratification within the study cohort (Figure 3D; Table S1).

### 3.4 Comparison and Evaluation of the Ischemic Growth Rate Model

**FIGURE 4:**
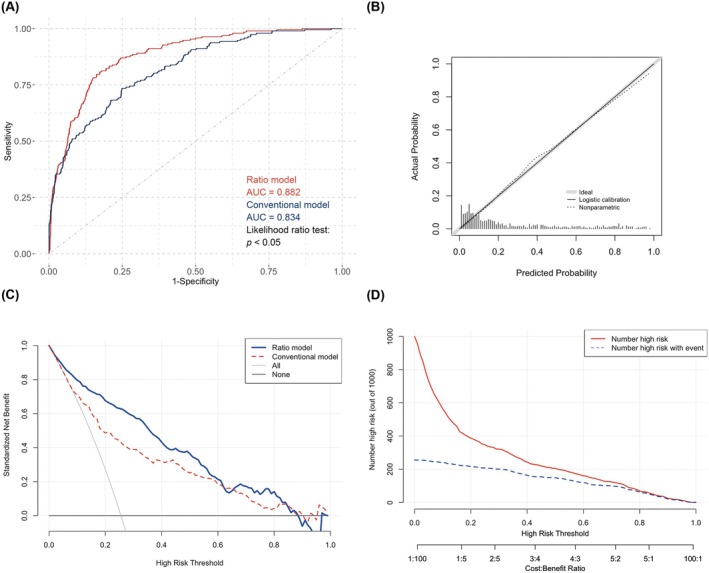
Evaluation of the growth rate model. (A) ROC curves of different models. (B) calibration curve of the growth rate model. (C) DCA of the growth rate and conventional models. (D) clinical impact curve of the growth rate model. AUC, area under the curve; DCA, decision curve analysis; Ratio model, the ischemic core growth rate model; ROC curve, receiver operating characteristic curve.

We have introduced a novel index termed “ischemic core growth rate,” which incorporates the consideration of OTT time into CTP information and developed a nomogram featuring it. To examine the predictive value of this growth rate model, we also developed a conventional index model that leverages conventional indices (Table S2) providing a benchmark against which the performance of the growth rate model could be evaluated. Subsequent comparative analysis revealed statistically significant difference between these two models (*p* < 0.05, likelihood ratio test). As both models included a same number of predictive factors (6 vs. 6), the growth rate model demonstrated a better fit (AIC: 574.6 vs. 642.8) and a better performance in discrimination (AUC: 88.2% vs. 83.4%) (Figure 4A). Besides, the calibration curve (bootstrap = 1000) displayed a good consistency between the actual occurrences and predicted probabilities within the study cohort (Figure 4B). Importantly, it also exhibited lower misclassification error (16.2% vs. 20.3%) [42]. These findings suggest that the growth rate model is superior to the conventional model in terms of model fitting and performing (Table S3). Furthermore, the DCA (Figure 4C) indicates that the growth rate model provides a higher net benefit across a threshold probability range of 0.1 to 0.8. Meanwhile, the clinical impact curve (Figure 4D) demonstrates a close proximity between the cases of a higher mRS score predicted by the model and the actual cases of poor prognosis at different threshold probabilities.

## 4 Discussion

Timely reperfusion of ischemic tissue represents a critical intervention for AIS [43]. A prediction model incorporating imaging data enables more comprehensive assessment and facilitates early therapeutic adjustment [44]. In our study, a high‐dimensional dataset with a total of 48 potentially predictive factors was employed. Next, we adopted a lasso‐logistic method for variable selection and model construction. Finally, six predictors were included in the nomogram: diabetes comorbidity, ischemic core growth rate, TOAST classification, neutrophil counts, DBIL level, and the NIHSS score at admission. This nomogram shows good performance in risk prediction and has the potential for clinical application.

The restricted therapeutic time window remains a significant barrier to reperfusion therapy for AIS [45, 46]. Classical studies indicate that IVT with alteplase maintains its safety and efficacy within 4.5 h after stroke onset [3]. However, emerging evidence suggests that IVT may still confer clinical benefits in select patients beyond the time window when guided by advanced imaging modalities to confirm a hypoperfused lesion‐ischemic core mismatch [17]. The EXTEND ClinicalTrails demonstrated that IVT administered from 4.5 to 9 h from last known well also improved the likelihood of favorable functional outcomes at 90 days compared with placebo [47]. These findings demonstrate that imaging‐based assessment of the tissue window has revised the rigid time‐based paradigm for IVT, thereby extending the reperfusion therapy eligibility for selected patients.

Advanced imaging modalities enable precise evaluation of the tissue window, with clinical studies demonstrating that both magnetic resonance imaging (MRI) and CTP provide reliable assessments of the penumbra and the ischemic core [48, 49]. In light of recent advancements in extending the time window of IVT, CTP imaging represents an increasingly recognized means of identifying patients who stand to benefit most from this therapy [50]. In addition, the predictive value of CTP imaging for functional outcome in stroke patients has garnered increasing attention from researchers in recent years [51]. Prior research employed CTP‐measured penumbra to predict 3‐month functional outcomes, with a convolutional neural network as the validation tool [52]. This finding exhibits the growing clinical utility of CTP imaging in prognosticating post‐thrombolysis functional outcomes. Our study further validates the prognostic significance of CTP‐derived ischemic core growth rate and demonstrates a strong correlation between this index and functional outcomes in AIS patients after IVT.

Besides IVT‐related parameters, our predictive model integrates additional critical predictors including diabetes comorbidity, TOAST classification, neutrophil counts, and DBIL levels, collectively achieving a superior discriminative performance (AUC: 0.882, 95% CI: 0.855–0.908). There is a close relationship between the etiological classification of stroke and prognosis. According to a follow‐up survey, patients with small artery occlusion have a lower probability of adverse outcomes compared to other types of stroke etiology, which is consistent with the study model in our research [53]. With diabetes prevalence rising globally, emerging evidence underscores its significant association with adverse outcomes in acute stroke patients [54]. In terms of pathophysiology, brain inflammation is recognized as one of the primary causes exacerbating secondary brain injury and impeding the prognosis of ischemic stroke [55]. A recent study confirms NE as pivotal therapeutic targets in ischemic stroke, with neutrophil extracellular traps inhibition alleviating ischemia–reperfusion injury in preclinical models [56]. Furthermore, research on the correlation between different bilirubin subtypes and adverse outcomes after IVT in patients with AIS indicates DBIL exhibits superior predictive accuracy compared to others [57]. Last but not least, there is a high predictive value of NIHSS scores for functional outcomes in stroke patients, as elevated scores are associated with poorer mRS outcomes at 3 months [34, 58]. Our study reaffirms the critical impact of these factors on predicting 3‐month functional recovery in AIS patients following IVT.

This study has several limitations. On the one hand, the retrospective nature of this study precluded the acquisition of complete data for all patients, such as collateral circulation status, which has demonstrated a correlation with outcomes of AIS patients in a previous study [28]. On the other hand, the absence of external validation limits the model's robustness and generalizability. Therefore, multicenter trials constitute a critical next step to confirm our findings through clinically diverse real‐world data. Additionally, calculation of the ischemic core growth rate index relies on advanced imaging processing software and exact time metrics, which may limit its clinical practice. Nevertheless, our model integrates novel clinical insights through robust statistical methods while maintaining concise implementation with strong predictive performance.

## 5 Conclusion

In this study, we developed a perspicuous nomogram featuring a novel ischemic core growth rate index to predict the neurological outcome of AIS patients using reliable statistical methods. Our model achieves accurate prediction with limited predictors, facilitating convenient clinical application. Through proactive identification and intervention, our model could help patients achieve a better outcome from this highly lethal and disabling disease.

## Ethics Statement

This study is approved by the Ethics Committee of the First Affiliated Hospital of Soochow University (No. 2024656) and the requirement of written informed consent was waived by the Ethics Committee.

## Conflicts of Interest

The authors declare no conflicts of interest.

## Supporting information


**Table S1:** cns70589‐sup‐0001‐TableS1.docx.

**Table S2:** cns70589‐sup‐0002‐TableS2.docx.

**Table S3:** cns70589‐sup‐0003‐TableS3.docx.

## Data Availability

The data that support the findings of this study are available on request from the corresponding author. The data are not publicly available due to privacy or ethical restrictions.
