# Supplementary material for: Intravenous Thrombolysis in Acute Ischemic Stroke: A Prognostic Prediction Model and the Role of Ischemic Core Growth Rate
Source: CNS Neurosci Ther. 2025 Sep 4;31(9):e70589. doi: 10.1111/cns.70589 (PMC12409076; doi:10.1111/cns.70589)
Supplement: Supplementary file 1 — Table S1: cns70589‐sup‐0001‐TableS1.docx. [file CNS-31-e70589-s002.docx]

SUPPLEMENTARY TABLE 1

Risk stratifications within the study cohort

| Risk_Stratification | Sub_category | case | Percentage |
| --- | --- | --- | --- |
| Low | Poor outcome | 38 | 7.70% |
| Low | Favorable outcome | 458 | 92.30% |
| High | Poor outcome | 160 | 62.70% |
| High | Favorable outcome | 95 | 37.30% |
