# Supplementary material for: Intravenous Thrombolysis in Acute Ischemic Stroke: A Prognostic Prediction Model and the Role of Ischemic Core Growth Rate
Source: CNS Neurosci Ther. 2025 Sep 4;31(9):e70589. doi: 10.1111/cns.70589 (PMC12409076; doi:10.1111/cns.70589)
Supplement: Supplementary file 2 — Table S2: cns70589‐sup‐0002‐TableS2.docx. [file CNS-31-e70589-s003.docx]

SUPPLEMENTARY TABLE 2

Independent variables included in the conventional model

| Variates | VIF | OR (95% CI) | *p* value |
| --- | --- | --- | --- |
| Ischemic core volume (mL) | 1.11 | 1.03 (1.02-1.04) | <0.001^***^ |
| History of ICH | 1.04 | 2.47 (1.34-4.59) | 0.004^**^ |
| Glu (mmol/L) | 1.03 | 1.08 (1.03-1.14) | 0.002^**^ |
| Tc (mmol/L) | 1.06 | 1.23 (1.04-1.49) | 0.020^*^ |
| Cr (μmol/L) | 1.01 | 1.01 (1.00-1.02) | 0.014^*^ |
| NIHSS score | 1.13 | 1.17 (1.13-1.22) | <0.001^***^ |

Abbreviations: VIF, variance inflation factor, a VIF < 5 usually indicates no obvious collinearity issue; OR, odds ratio; CI, confidence interval; ICH, intracranial hemorrhage; Glu, glucose; Tc, total cholesterol; Cr, creatinine; NIHSS, National Institutes of Health Stroke Scale score.

^*^ Indicates a *p* value < 0.05.

^**^ Indicates a *p* value < 0.01.

^***^ Indicates a *p* value < 0.001.
