# Supplementary material for: Intravenous Thrombolysis in Acute Ischemic Stroke: A Prognostic Prediction Model and the Role of Ischemic Core Growth Rate
Source: CNS Neurosci Ther. 2025 Sep 4;31(9):e70589. doi: 10.1111/cns.70589 (PMC12409076; doi:10.1111/cns.70589)
Supplement: Supplementary file 3 — Table S3: cns70589‐sup‐0003‐TableS3.docx. [file CNS-31-e70589-s001.docx]

SUPPLEMENTARY TABLE 3

Evaluation and comparison between the ratio model and conventional model

| Model | Variable inclusion method | Number of variables | AIC | Misclassification error (%) | AUC (95% CI) | Hosmer-Lemeshow test (*p* value) | R^2^ | Likelihood ratio test (*p* value) |
| --- | --- | --- | --- | --- | --- | --- | --- | --- |
| Ratio model | Lasso-logistic method | 6 | 574.6 | 16.22 | 0.882 (0.855-0.908) | 0.851 | 0.487 | 0.046^*^ |
| Convention model | Lasso-logistic method | 6 | 642.8 | 20.25 | 0.834 (0.802-0.867) | 0.438 | 0.415 |  |

Abbreviations: Ratio model, the ischemic core growth rate model; AIC, Akaike Information Criterion, a lower AIC indicates a better model fit; AUC, area under the curve; CI, confidence interval; R^2^, coefficient of determination, a higher R^2^ indicates a bitter model fit.

^*^ Indicates a *p* value < 0.05
